# Machine learning for contour classification in TG‐263 noncompliant databases

**DOI:** 10.1002/acm2.13662

**Published:** 2022-06-10

**Authors:** David Livermore, Thomas Trappenberg, Alasdair Syme

**Affiliations:** ^1^ Department of Physics and Atmospheric Science Dalhousie University 6299 South St Halifax NS B3H 4R2 Canada; ^2^ Faculty of Computer Science Dalhousie University 6050 University Avenue Halifax NS B3H 4R2 Canada; ^3^ Department of Radiation Oncology Dalhousie University 5820 University Avenue Halifax NS B3H 1V7 Canada

**Keywords:** image classification, machine learning, standardization

## Abstract

A large volume of medical data are labeled using nonstandardized nomenclature. Although efforts have been made by the American Association of Physicists in Medicine (AAPM) to standardize nomenclature through Task Group 263 (TG‐263), there remain noncompliant databases. This work aims to create an algorithm that can analyze anatomical contours in patients with head and neck cancer and classify them into TG‐263 compliant nomenclature. To create an accurate algorithm capable of such classification, a combined approaching using both binary images of individual slices of anatomical contours themselves, as well as center of mass coordinates of the structures are input into a neural network. The center of mass coordinates were scaled using two normalization schemes, a simple linear normalization scheme agnostic of the patient anatomy, and an anatomical normalization scheme dependent on patient anatomy. The results of all of the individual slice classifications are then aggregated into a single classification by means of a voting algorithm. The total classification accuracy of the final algorithms was 97.6% mean accuracy per class for nonanatomically normalization scheme, and 97.9% mean accuracy per class for anatomically normalization scheme. The total accuracy was 99.0% (13 errors in 1302 structures) for the nonanatomically normalization scheme, and 98.3% (22 errors in 1302 structures) for the anatomically normalization scheme.

## 1 INTRODUCTION

Radiation therapy is a treatment modality that is indicated for many oncologic malignancies and some benign functional disorders. It is a technologically‐advanced field of medicine that is process‐oriented. As such, there are many steps in the treatment workflow that are amenable to machine learning (ML) methods. Examples of applications of ML methods from the recent literature include: automated diagnosis,^[^ ^1^ ^]^ tissue segmentation,^[^ ^2^ ^]^ treatment planning^[^ ^3^, ^4^ ^]^, and outcome prediction^[^ ^5^ ^]^.

The quality of data in a radiation therapy is high: high‐resolution CT images, expert‐drawn tissue contours, and high‐accuracy dose distribution calculations. The volume of high quality across modern radiation therapy centers around the world is immense. Tools to permit the mining of these data would be of great benefit to investigators interested in applying ML methods to challenges in radiation therapy. However, significant barriers exist that prevent this from happening currently. Barriers related to data confidentiality are obviously very important, but are not within the scope of the current work. Instead, this work proposes a solution to a technical challenge that has very significant implications for data mining efficiency: structure classification.

### 1.1 Problem Statement and hypothesis

Modern ML algorithms can often require a large amount of data to train properly, and acquiring this data can be difficult, particularly with medical data. The lack of standardization further exacerbates this, as a particular structure may have different labels. For example, the left parotid gland may be labeled “Left Parotid,” or “Parotid_Left,” “Parotidl,” etc. There exist numerous public databases of medical data, such as the Cancer Imaging Archive,^[^ ^6^ ^]^ and while these do contain numerous image datasets, labeling of regions of interest (ROIs) and organs at risk (OARs) remains inconsistent between the various datasets. Thus, there exists a demand for a method of curating the data.^[^ ^7^ ^]^ The American Association of Physicists in Medicine (AAPM) through Task‐Group 263 (TG‐263) sought to improve this by standardizing nomenclature for ROIs and dose‐volume histogram (DVH) metrics.^[^ ^8^ ^]^ This standardization was in response to the development of TG‐113 in which standardization of clinical trial methodologies recommended standardization of nomenclature to facilitate data pooling between clinical trials.^[^ ^8^, ^9^ ^]^ Moving forward, institutes that choose to use the TG‐263 standard could potentially share data, but could lead to the exclusion of datasets that predate TG‐263, as well as data from institutes that choose not to comply with TG‐263.

### 1.2 Contributions and related work

Efforts to classify already contoured structures are often made in the context of error detection. McIntosh et al. (2013) used a Groupwise Conditional Random Forest (GCRF) approach for detection of erroneously labeled structures located in the chest, abdomen, and pelvis, achieving an accuracy for organs at risk of 97% and accuracy for target volumes of 85%.^[^ ^10^ ^]^ This method was chosen as the authors believed that CNNs lacked the ability to discriminate similar shapes with a sufficient level of accuracy.

Altman et al. (2015) used a series of metrics of the contours themselves, such as the number of slices the structure appears on, and axial area to construct a database of metrics associated with known good contours; achieving a sensitivity of 0.95 and specificity of 0.81, respectively.^[^ ^11^ ^]^

Chen et al. built a similar geometric attribute distribution (GAD) that characterized a contour's mechanical properties, such as shape and centroid, and compare it against other structures, achieving sensitivity and specificity of up to 1 and 0.979, respectively, for training set, with sensitivities between 0.848 and 0.908, and specificities between 0.824 and 0.837 on the test set.^[^ ^12^ ^]^

Altman et al. and Chen et al. both studied nine structures: brain, brainstem, left and right eyes, left and right optic nerves, left and right parotids, and optic nerve. In this work, we analyze all of the aforementioned structures, as well as the left and right cochlea, left and right lenses, esophagus, spinal cord, larynx, and pituitary gland (see Table 1).

**TABLE 1:** Summary of regions of interest (ROI) along with the TG‐263 compatible name, the mean number of slices per patient that contain such ROI, and the standard deviation of the mean

ROI	TG‐263 name	Train set (slices)	Test set (slices)
Left eye	“Eye_L”	4788	1101
Right eye	“Eye_R”	4738	1090
Left cochlea	“Cochlea_L”	161	53
Right cochlea	“Cochlea_R”	158	55
Larynx	“Larynx”	5750	1283
Left lens	“Lens_L”	1504	348
Right lens	“Lens_R”	1494	345
Chiasm	“Chiasm”	810	196
Left optic nerve	“OpticNerve_L”	1125	281
Right optic nerve	“OpticNerve_R”	1086	278
Brainstem	“Brainstem”	11 536	2567
Brain	“Brain”	25 590	5727
Esophagus	“Esophagus”	20 832	4824
Spinal cord	“SpinalCord”	21 746	4824
Left parotid	“Parotid_L”	10 031	2220
Right parotid	“Parotid_R”	9879	2183
Pituitary gland	“Pituitary”	1357	298
Total		122 582	22 709

Although an application of the algorithm discussed in this paper could be used in error detection, we will discuss it in the more general case of contour classification.

In this work, we will introduce a novel tool that uses a convolutional neural network (CNN) based on the ResNet18^[^ ^13^ ^]^ architecture to analyze the contours of a particular patient CT slice and classify that contour. Analyzing the contours of many slices, we can aggregate the classifications and assign a classification based on a consensus.

One of the challenges associated with working with medical data is the inherent unbalanced nature of datasets. In this work, structures to be classified have been drawn on CT datasets. Because these images are sliced axially, certain ROIs are inherently overrepresented. Patients tend to have far more images with a spine contour as compared to ROIs like the eyes or optic chiasm. Although we considered several methods for handling data imbalance, ultimately, we decided to increase the weights on the most challenging ROIs, the cochlea, and pituitary gland (see Table 3). Preliminary evaluation of network performance suggested that increased the relative weights of several ROIs improved overall network performance (data not shown).

We describe an algorithm that is capable of automatically classifying ROIs. The approach we use is unique as we combine images of the segments of the ROIs that we wish to classify, as well as the position and geometric properties of the contour itself to inform our classification algorithm. Combining these different data together is quite important to overcome some of the downsides of each individual classification method. Using an image of the segment of the ROI, a CNN can easily classify ROIs with different shapes. However, it is very difficult for CNNs to differentiate objects of similar shapes, at different positions, such as the left eye from the right eye, or the esophagus from the spine.^[^ ^10^ ^]^ Using the position of the ROI, one can easily tell the difference between two objects that are consistently separated in space, such as the left and right eye, but will struggle with ROIs that about one another, such as the spine from the brainstem.^[^ ^10^ ^]^ We can then compile the results for numerous slices of the same ROI to evaluate how each slice of the ROI is identified.

One of the differences between this work and previously published work described earlier is that previously published work attempts to classify the entire structure. This work breaks each structure into slices, attempts to classify individual slices, and then aggregates the results of the many classifications into a single classification of the entire ROI. Although we believe that this work could be modified to perform classification on whole structures using three‐dimensional classifier, this would require a significantly larger memory capacity as compared to a two‐dimensional classifier. Thus, it was decided for this work to limit the work to two dimensions.

Ultimately, this work will help facilitate the automated mining of historical and current databases to enhance the efficiency of ML research in those applications in which accurate identification of tissue structures is of importance.

## 2 METHODS AND MATERIALS

This work is a retrospective study that received approval from the local ethics board. CT image and contour data from 546 previously treated head and neck cancer patients were anonymized and exported from the Eclipse treatment planning system using an in‐house‐developed ESAPI script. All OARs were contoured by experienced, CMD‐certified dosimetrists and approved by a radiation oncologist. Of the 546 data sets, all patients contoured before TG‐263 were labeled with an in‐house‐standardized nomenclature; patients after TG‐263 were labeled with a TG‐263 compliant scheme. Each CT image had a size of 512 × 512 voxels and images were not preprocessed to match voxel dimensions, with a uniform slice thickness of 2.5 mm.

Contours were saved in loss‐less compressed NumPy^[^ ^14^ ^]^ arrays. Of the 546 patients, 100 were withheld to serve as a test group. The remaining 446 served as a training set, 80% of which were assigned to a train group and 20% were assigned to a validation group. Table 1 outlines a brief summary of the patient data used to train the network. Note that there is a small asymmetry between certain classes, this can be attributed to several factors include asymmetry in the patient anatomy, or partial volume effects within the CT that affected the delineation of contours.

In this work, we evaluate three different algorithms; the first using only information regarding the position of the segment along the axial plane (Section 2.1), the second using only a fully CNN to classify images of the segments (Section 2.2), the third is an algorithm that incorporates both positional data, and binary images of the contour to perform classification (Section 2.3).

The computer used to perform the calculations is a desktop computer running Ubuntu Linux 20.04.2 LTS, Intel Core i5‐6500 3.2 GHz processor, 16 GB of RAM, using GeForce RTX 2070 GPU with 8 GB of RAM for network training.

### 2.1 Position‐based classifier

**FIGURE 1:**
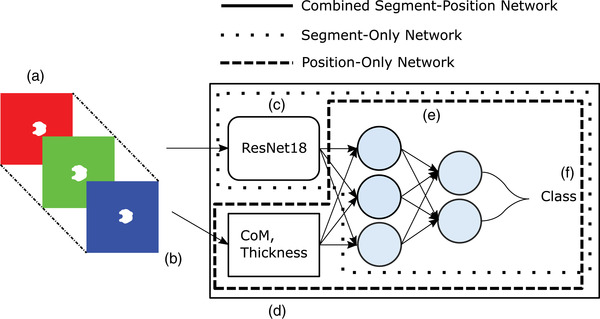
Full network diagram for the combined segment‐position‐based classifier. (a) Input stack of images showing the slice to be classified in the center, with preceding and succeeding images above and below, respectively (example slices are from a brain). (b) The images are “stacked” into a three‐dimensional array, of shape 3×512×512 and input into the pretrained ResNet18 (c). (d) The center‐of‐mass (CoM) is extracted from the slice to be classified and input into the dense neural network alongside the output of the ResNet18 (e). (f) The output of the network is a vector whose elements are probabilities that the slice in question is assigned to each class

The first network evaluated was a simple dense, or fully connected, artificial neural network (see Figure 1, “Position‐Only Network”). Several versions were tested including ones with and without a hidden layer, but in all cases, the input is a tuple of scalars, and the output is a vector of probabilities associated with each class. Contour points were converted into binary masks (1 inside the contour and 0 outside) and the geometric center of the mask for each contour on each image slice was calculated. To test whether or not a pixel should be a 1 or 0, the center point of the pixel was checked to see if it was inside the contour. No subsampling or supersampling methods were utilized.

Images that are 512 by 512 pixels yield coordinates pairs of integer value between 0 and 511; however, most of the contours are localized around the center of the image. As such in addition to unnormalized *x*–*y* coordinate pairs, two normalization schemes were studied. In the first normalization scheme, all coordinates were linearly scaled from [0, 511] to [ − 1, 1] in both the *x* and *y* directions in the axial plane. Coordinates along the *z* direction (cranial‐caudal) were also normalized by taking the slice index of the superior slice of the patient's brain and setting that coordinate to be 1, and the inferior slice of the patient's brain to be 0, and then linearly mapping the slice index of each slice for a patient accordingly. In the second normalization scheme, the left‐ and right‐most extremes of the patient's brain contour were identified and assigned coordinates of −1 and 1, respectively, and all other coordinates scaled linearly. This was repeated for the anterior‐posterior direction, setting the posterior‐most point of the brain to be 1, and anterior‐most point to be −1. For the *z*‐direction, the most superior point was assigned a coordinate of 1, and the most inferior point −1.

The choice of brain for coordinate normalization was arbitrary, but justified based on the facts that: (1) it was contoured on all patients in our dataset and (2) it is large enough that small differences in contouring or voxelization should be minimal.

All position‐based classification that included the *z*‐axis also included *z*‐axis normalization as the range of possible values depended on the positioning of the patient within the scanner and ranges for possible values varied dramatically.

### 2.2 Segment‐based classifier

The second algorithm employed a transfer learning approach,^[^ ^15^ ^]^ using a ResNet18 and^[^ ^13^ ^]^ pretrained on ImageNet (see Figure 1, “Segment‐Only Network”).^[^ ^16^ ^]^ The ImageNet‐trained ResNet18 is expecting an input with three channels, corresponding to the red, green, and blue color channels; in this work, we input a single slice binary mask of the ROI we wish to contour into the “green” channel. In a single‐slice classification scheme, we simply fill the other two channels with 0's. In a multislice scheme, we fill the adjacent slices into the “red” and “blue” channels (if the slices exist, otherwise leave as zeros). We remove the last layer of the ResNet18, and replace it with a hidden layer, followed by a fully connected output layer. This was done to match the number of outputs to the number of classes.

Previous work by Xu et al. has demonstrated that combining consecutive slices dramatically improves the performance of CNNs in medical imaging applications.^[^ ^17^ ^]^

### 2.3 Combined segment–position‐based classifier

Using the same methods as described in Section 2.1, we generated center‐of‐mass coordinates from binary masks of contour images. The inputs to our network are then the binary image of the contour (input into a ResNet18), as well as the center‐of‐mass, which are concatenated to the final layer of the ResNet18 (see Figure 1 “Combined Segment‐Position Network”). Similarly to work in Section 2.2, the number of nodes in the hidden layer was determined using a Bayesian hyperparameter selection using a Tree‐structured Parzen Estimator in Optuna.^[^ ^18^, ^19^, ^20^ ^]^

Three versions of this classifier were tested: the first with position coordinates normalized to [−1,1] (nonanatomical coordinates), one with position coordinates normalized anatomically as described in Section 2.1, and a second network anatomically normalized networked trained using the eyes and brainstem to provide cradial‐caudal and lateral extent to define the [−1,1] normalization points. This normalization scheme was considered since it is not necessarily standard clinical practice to include the entirety of the head in the planning CT scan. The alternative anatomical normalization using the eyes and brainstem was only tested for the combined segment and position‐based classifier. This is because, as it will be shown in the results, the combined classifier produced better results compared to the position only classifier.

In addition to the inclusion of a coordinate system, as a further input to the network, we included the total number of slices in the ROI being identified. For this dataset, which uses uniform slice thickness of 2.5 mm, the number of slices will be related linearly to the length of the ROI along axial direction. This information was included as early trials of our network demonstrated that including additional information had a dramatic effect on network performance (see Section 3.1).

### 2.4 Voting algorithm

The output of each network studied in Sections 2.1, 2.2, 2.3 was a slice‐by‐slice classification of each contour present on a given slice. No restrictions were placed on the output of the networks, meaning that more than one contour on a given slice could be assigned to the same class. Ultimately, the output of the networks was used to assign a class to an entire ROI (composed of multiple contours on multiple slices). For each slice in a structure, we perform classification on the given slice and consider the class selected by the algorithm as a “vote” in favor of that particular structure. The structure with the most overall votes is considered the winner.

Owing to the large number of networks described in the methods, Table 2 has been provided with a brief summary with an abbreviated nomenclature scheme, which will be referred to in all subsequent text.

**TABLE 2:** Summary of networks tested

Network	Description
PU	Position along axial plane only, unnormalized
PU‐32	Position along axial plane only, unnormalized, with 32‐node hidden layer
PN	Position along axial plane only, normalized (nonanatomical)
PN‐32	Position along axial plane only, normalized (nonanatomical), with 32‐node hidden layer
PN‐32Z	Position in three dimensions, normalized (nonanatomical), with 32‐node hidden layer
PA‐32Z	Position in three dimensions, anatomically normalized, with 32‐node hidden layer
SS	Segmentation‐only using a single slice image
SM	Segmentation‐only using three consecutive images
CNS	Combined position‐segmentation classifier, normalized (nonanatomical), slice‐by‐slice classification
CNV	Combined position‐segmentation classifier, normalized (nonanatomical), after application of the voting algorithm
CAS‐B	Combined position‐segmentation classifier, anatomically normalized to the brain, slice‐by‐slice classification
CAS‐E	Combined position‐segmentation classifier, anatomically normalized to the eyes and brainstem, slice‐by‐slice classification
CAV‐B	Combined position‐segmentation classifier, anatomically normalized to the brain, after application of the voting algorithm
CAV‐E	Combined position‐segmentation classifier, anatomically normalized to the eyes and brainstem, after application of the voting algorithm

### 2.5 Robustness testing

To test the robustness of the network against missing slices of contoured structures, 34 patients were selected at random from the test set. For each patient, a single organ was selected and a single slice from the organ was removed from the dataset with each organ being selected exactly twice. The remaining slices were then classified using our network, with arrays of zeroes filling in the missing slices. In this instance, the entire structure is classified slice‐by‐slice as per section 2.3 using the brain anatomically normalized network, and then the voting algorithm is applied to create a single classification as per Section 2.4.

## 3 RESULTS

### 3.1 Position‐based classifier

**FIGURE 2:**
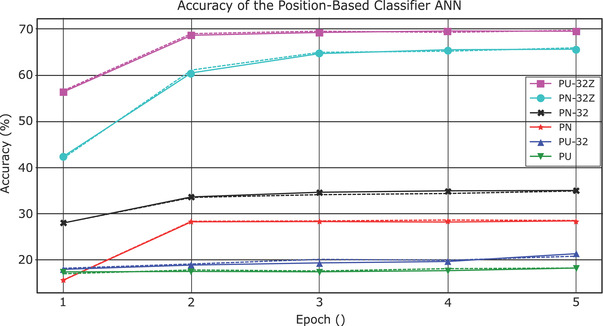
Mean accuracy of the position‐based classifier as a function of number of training epochs. (Training accuracy are in solid lines, validation accuracy in dashed lines)

Results for the position‐based classifier are sufficiently inferior that only the total accuracy statistics (i.e., percentage of correctly classified contours out of the total number of contours in the test set) on a slice‐by‐slice basis are reported. Figure 2 shows the performance of all of the position‐based classifiers described in Section 2.1. Total accuracy is plotted as a function of epoch. Unnormalized coordinates with no hidden layers (PU, green) achieved a total accuracy of 18.1% in the final epoch on the validation set. The inclusion of a 32‐node hidden layer (PU‐32, blue) only improved the results to 21.1%. For perspective, guessing randomly with equal probability of each class would result in total accuracy of 5.8%, and guessing randomly with probability proportional to the number of slices of each class would achieve a total accuracy of 13.1%. Normalizing the coordinates, without including a hidden layer improved the total accuracy to 28.4% (PN, red); adding a hidden layer with the normalized coordinates improved the total accuracy to 34.8% (PN‐32, black). Including the normalized *z* coordinates with a 32‐node hidden layer improved the classification accuracy to 65.5% (PN‐32Z, cyan). Using anatomical normalization of the coordinate system improved total accuracy to 69.5% (PA‐32Z, magenta).

### 3.2 Segmentation‐based classifier

**FIGURE 3:**
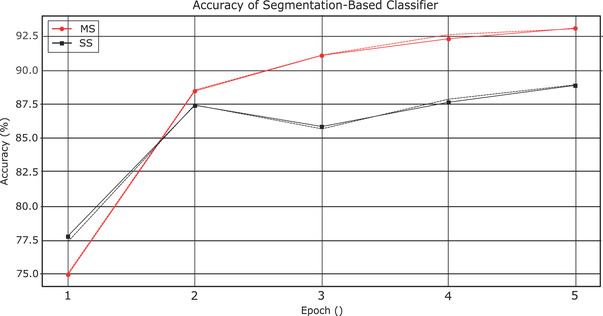
Plot of the accuracy of SS versus SM classifier networks. (Training accuracy are in solid lines, validation accuracy in dashed lines)

Figure 3 shows the total accuracy as a function of epoch when the classification is based on contour segmentation. The benefit of including adjacent image slices in the red and blue channels becomes apparent after the first epoch. The total accuracy of the multislice classifier (SM) is 93.0% on the final epoch compared with 88.8% with the single‐slice classifier (SS). Classification based on segmentation alone is superior to classification based on position alone, which only achieved a total accuracy of at most 69.5%.

### 3.3 Combined segment–position‐based classifier

**FIGURE 4:**
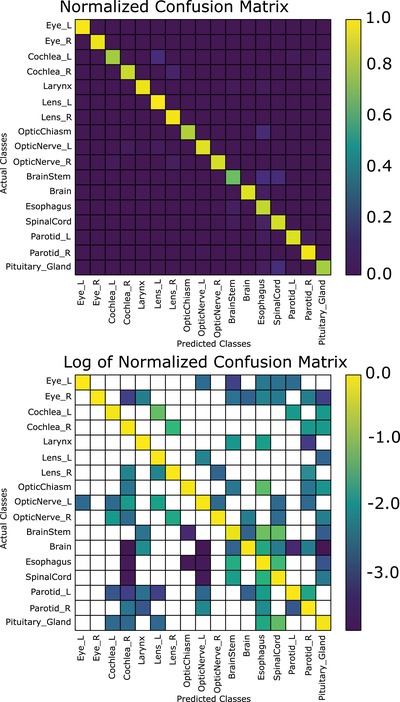
Normalized confusion matrix (top) and log (base‐10) of normalized confusion matrix for the slice‐by‐slice classification scheme, nonanatomical normalized coordinates (blank areas indicate zeroes)

**FIGURE 5:**
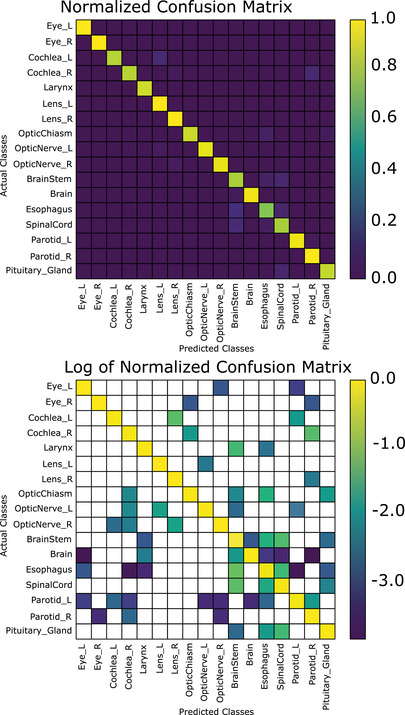
Normalized confusion matrix (top) and log (base‐10) of normalized confusion matrix for the slice‐by‐slice classification scheme, brain anatomically normalized coordinates (blank areas indicate zeroes)

The combined segmentation and position‐based classifier achieved a mean accuracy per class (i.e., the proportion of correctly identified structures averaged over all structures) of 92.0% when using the first (nonanatomical) normalization scheme (CNS); while mean accuracy per class was 93.3% when using the brain anatomical normalization scheme (CAS‐B). In terms of total accuracy, CNS achieves a total accuracy of 97.0%, while CAS‐B achieves a total accuracy of 97.3%, approximately 4% better than the best segmentation‐based classifier (Figure 5). Figure 4 demonstrates the confusion matrix of our slice‐by‐slice algorithm, normalized such that the sum of each column is 1 (top), and the log base‐10 of the confusion matrix on the bottom. Normalizing the coordinates to the eyes and brainstem (CAS‐E) as opposed to the brain had a slight reduction in the total accuracy on both a slice‐by‐slice and after the voting algorithm. The mean accuracy per class was 91.6% when normalizing to the eyes and brainstem. A brief summary of these results is presented in Table 4. It is worth noting that the spinal cord classification accuracy was low when using the eyes and brainstem normalization (71.6%, see Table 7); without including the spine, the mean accuracy per class is 92.9%.

Hyperparameters of the final network were determined using Bayesian Hyperparameter search using a Tree‐Parzen Estimator in Optuna.^[^ ^18^, ^19^, ^20^ ^]^ The final hyperparameters used in the network are detailed in Table 3.

**TABLE 3:** Summary of hyperparameters used in network design and training (Dropout 1 denotes dropout between the concatenated output of the ResNet18 and the hidden layer; dropout 2 denotes the dropout between the hidden layer and the output layer.)

Hyperparameter	Value
Batch size	12
Learning rate	0.1
Learning rate decay	0.5
Weight decay (L_2_)	0
Dropout 1^†^	0.6
Dropout 2^†^	0.2
Size of hidden layer	64
Cochlea weight	10
Pituitary weight	10
All other class weights	1

### 3.4 Voting algorithm

**FIGURE 6:**
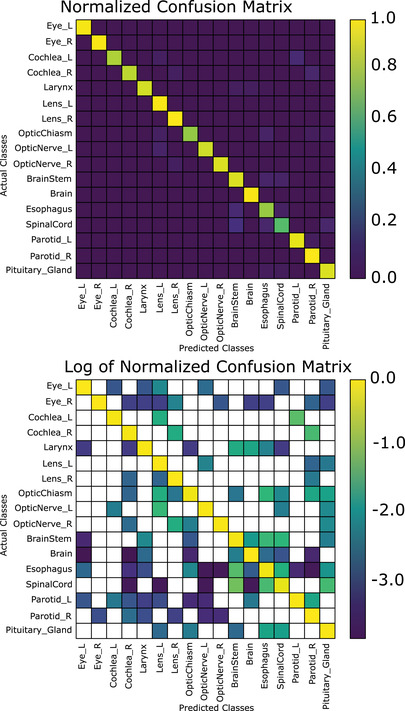
Normalized confusion matrix (top) and log (base‐10) of normalized confusion matrix for the slice‐by‐slice classification scheme, using the eyes and brainstem normalized coordinates (blank areas indicate zeroes)

**FIGURE 7:**
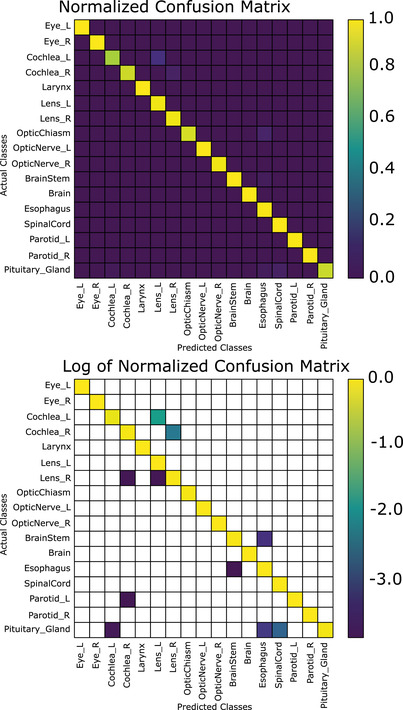
Normalized confusion matrix (top) and log (base‐10) of normalized confusion matrix for the structure classification scheme after voting, non‐anatomically normalized coordinates (blank areas indicate zeroes)

**FIGURE 8:**
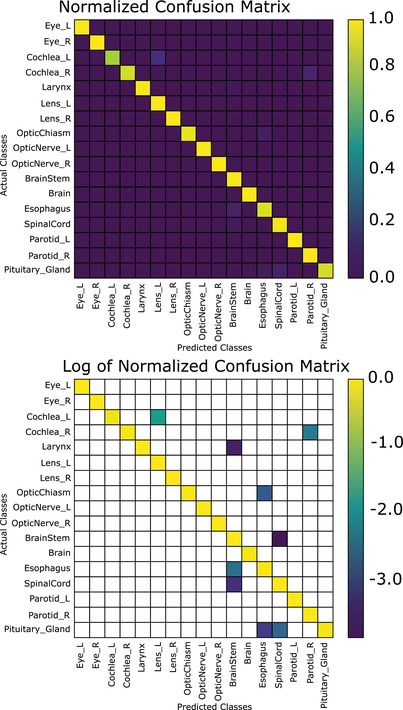
Normalized confusion matrix (top) and log (base‐10) of normalized confusion matrix for the structure classification scheme after voting, brain anatomically normalized coordinates (blank areas indicate zeroes)

**FIGURE 9:**
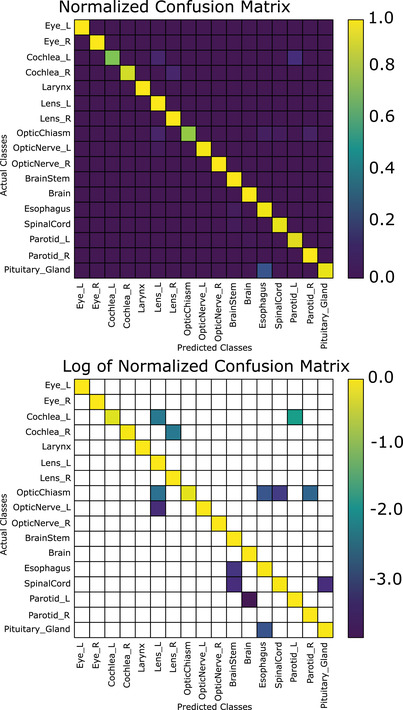
Normalized confusion matrix (top) and log (base‐10) of normalized confusion matrix for the structure classification scheme after voting, eyes and brainstem anatomically normalized coordinates (blank areas indicate zeroes)

Using the voting algorithm, we are able to achieve an total accuracy of 99.0% (13 errors in 1302 structures; mean accuracy per class of 97.6%) when using the nonanatomical coordinates (CNV), 98.3% (22 errors in 1302 structures, mean accuracy per class of 98.1%) when using the brain anatomical coordinates (CAV‐B); and 97.9% (27 errors in 1302 structures, mean accuracy per class 96.7%) when using the eyes and brainstem anatomical normalization coordinates (CAV‐E) (Figure 6). In Figure 7, the normalized and log‐10 confusion matrices demonstrate only a handful of incorrect classifications for the brain‐normalized network. Similarly, Figure 8 shows the normalized and log‐10 confusion matrices for the anatomical model using the brain normalization scheme. Figure 9 demonstrates the normalized and log‐10 confusion matrices for the anatomical model using the eyes‐brainstem normalization scheme. While the overall number of errors in the network is reduced when using the nonanatomical coordinates, the number of misclassified cochlea is reduced when using anatomical coordinates.

**FIGURE 10:**
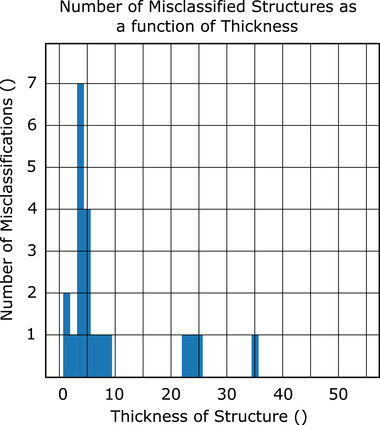
Histogram of number of classification failures as a function of number of slices in a structure to be classified, nonanatomical coordinates

**FIGURE 11:**
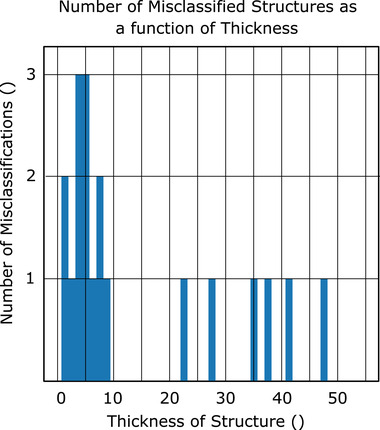
Histogram of number of classification failures as a function of number of slices in a structure to be classified, brain anatomical coordinates

**FIGURE 12:**
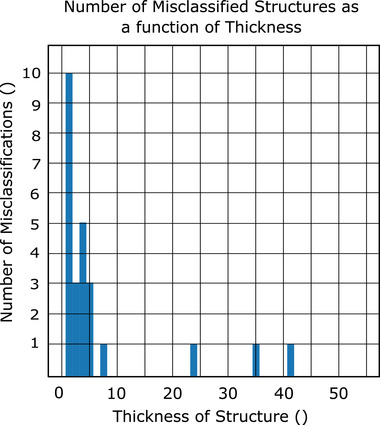
Histogram of the number of classification failures as a function of the number of slices in a structure to be classified, eyes and brainstem anatomical coordinates

In Figures 10, 11, 12, histograms of the number of failed classifications as a function of structure thickness (i.e., number of image slices containing the incorrectly classified ROI) are shown for the cases of nonanatomical coordinates (Figure 10) and brain anatomical coordinates (Figure 11) and eyes and brainstem anatomical coordinates (Figure 12). A majority occur for structures with fewer than 10 slices.

Table 4 provides a brief summary of the difference between CNS, CNV, CAS‐B, and CAV‐B. Table 5 shows a more detailed breakdown of the total accuracy of CNS, and CNV networks on each of the anatomical structures. Table 6 shows a more detailed breakdown of the accuracy of CAS‐B, and CAV‐B networks on each of the anatomical structures.

**TABLE 4:** Summary of results for the nonanatomically normalized coordinates and anatomically normalized coordinates for both the mean accuracy per class on a slice‐by‐slice basis, and after applying the voting algorithm (all values are states as mean accuracy per class)

Method	Slice accuracy (%)	Voting (ROI) accuracy (%)
Nonanatomically Coords, CNS, CNV	92.0	97.6
Brain Anatomically Coords, CAS‐B, CAV‐B	93.3	97.9
Eyes and Brainstem Anatomically Coords, CAS‐E, CAV‐E	91.6	96.7

**TABLE 5:** Summary of the accuracy for individual ROIs using CNS and CNV

ROI	Slice accuracy (%)	Voting accuracy (%)
Left eye	98.2	100.0
Right eye	96.3	100.0
Left cochlea	84.9	86.7
Right cochlea	90.9	93.3
Larynx	96.1	100.0
Left lens	98.9	100.0
Right lens	97.2	97.8
Optic chiasm	87.2	94.1
Left optic nerve	93.9	100.0
Right optic nerve	93.0	100.0
Brainstem	75.2	98.0
Brain	94.0	100.0
Esophagus	89.6	98.9
Spinal cord	92.5	100.0
Left parotid	93.8	98.9
Right parotid	97.1	100.0
Pituitary gland	85.2	92.0
Mean	92.0	97.6

**TABLE 6:** Summary of the accuracy for individual ROIs using CAS‐B and CAV‐B

ROI	Slice accuracy (%)	Voting accuracy (%)
Left eye	99.7	100.0
Right eye	99.6	100.0
Left cochlea	88.7	93.3
Right cochlea	89.1	93.3
Larynx	93.7	98.8
Left lens	99.4	100.0
Right lens	99.4	100.0
Optic chiasm	91.6	96.5
Left optic nerve	95.9	100.0
Right optic nerve	96.5	100.0
Brainstem	86.9	99.0
Brain	97.5	100.0
Esophagus	80.5	95.7
Spinal cord	86.1	96.2
Left parotid	97.2	100.0
Right parotid	99.6	100.0
Pituitary gland	91.0	91.0
Mean	93.6	97.9

**TABLE 7:** Summary of the accuracy for individual ROIs using CAS‐E and CAV‐E

ROI	Slice accuracy (%)	Voting accuracy (%)
Left eye	98.0	100.0
Right eye	98.2	100.0
Left cochlea	86.8	80.0
Right cochlea	89.1	93.3
Larynx	92.7	100.0
Left lens	98.3	100.0
Right lens	98.9	100.0
Optic chiasm	82.8	83.5
Left optic nerve	91.8	100.0
Right optic nerve	94.1	100.0
Brainstem	84.9	100.0
Brain	94.4	100.0
Esophagus	83.5	97.9
Spinal cord	71.6	96.2
Left parotid	95.5	98.9
Right parotid	99.2	100.0
Pituitary gland	93.9	96.6
Mean	91.6	96.7

### 3.5 Robustness testing

The algorithm proved to be quite robust against small gaps in the slices of the structures. Out of 34 organs evaluated, 32 were correctly identified when there were no missing slices, and 30 were correctly identified when there was a single missing slice. When there were no missing slices, the two errors being an esophagus classified as a brainstem, and an optic chiasm classified as an esophagus. When there were missing slices, the previously mentioned errors persisted, and the two new misclassifications were a right eye mislabeled as a brainstem, and a left cochlea being mislabeled as a left lens. In the instance of the first misclassification, six slices were initially classified right eye, five slices were classified brainstem, and one was right lens; and in the instance with missing slices, four were labeled right eye, five were labeled brainstem, and two were labeled right lens. In the case of the misclassified cochlea, initially two slices were labeled left cochlea and one slice was labeled left lens, and after removing a slice, one slice was classified as a left cochlea and one slice was classified as a left lens, leading to a tie (ties are considered misclassification).

## 4 DISCUSSION

Normalization of the coordinate system plays a role in ensuring that a network is able to classify structures. We attribute this to two factors: centering the coordinate system in the middle of the field allows the algorithm to more easily differentiate between left and right anatomy; and a normalization reduces the absolute differences between training values. Using the anatomical coordinates also had an improvement for several structures. This is likely due to the fact that patient anatomy can vary quite a bit, but each patient will likely have similar proportions in their anatomy.

Using multiple slices of contour data results in a marked improvement in the performance of the network in the segmentation‐based approach since this affords the classification algorithm more information. It is important to note that the position‐based and segment‐based classifiers were only trained for five epochs and with limited hyperparameter tuning. This was intentional as these networks were selected to demonstrate the relative impact of normalization and utilization of multiple slice data in network performance. The training and testing curves also appear to be quite similar, which may be because the training set was sufficiently large that it was representative of the anatomical distribution contained within the test set.

The voting algorithm used in this paper is a simple approach to electoral systems. There are numerous other possible voting systems (see, e.g.,^[^ ^21^ ^]^), and future work could determine a more suitable approach for classification problems. We considered several alternatives, including a ranked‐ballot approach, but found that in many of the cases that challenge our network, it did not make a meaningful difference, and thus opted to keep the first‐past‐the‐post method.

A nuanced difference between the anatomical and nonanatomical normalization schemes is the accuracy for the individual classes. For example, in Figure 7, the nonanatomical normalization scheme achieves 100% accuracy on 11 out of 17 classes; three other classes achieving an accuracy of greater than 95%. The largest proportion of errors can be attributed to the left and right cochlea as well as the pituitary. In Figure 8, 8/17 structures have 100% accuracy, and five more are at least 95% or greater. The left and right cochlea achieving only 93.3% and 86.7% accuracy, respectively, and the pituitary achieving 93.3% responsible for a large proportion of the errors. Thus, while the brain anatomical normalization achieves better mean accuracy per class as compared to the nonanatomical normalization, it has fewer classes with 100% accuracy.

All normalization schemes yield algorithms that struggle to perform classification on structures with a small number slices (typically less than 10) after the voting process (see Figures 10, 11, 12). As an extreme example, classification of the brainstem in CNS algorithm was 75.2% accurate on a slice‐by‐slice basis, but after CNV was 98.0% accurate. Compared to the left cochlea for the same algorithm, only 84.6% accuracy on a slice‐by‐slice basis, and after voting, this only improves to 86.7%. This is due to the fact that for a large organ, it is unlikely that a majority of slices will be misclassified, but if the number of slices is very small, as in the case of the cochlea, the probability of misclassification is still high. Among the structures with a larger number of slices, misclassification generally occurs between structures with similar shape placement along the axial plane; the pituitary gland, for example, is often misclassified in CAV‐B, CAV‐E, and CNV as either an esophagus or spinal cord.

Although the anatomical normalization scheme presented here used the brain as the reference anatomy, it is likely that both anatomical and nonanatomical versions of these algorithms would generalize to other anatomical sites, provided that a suitable reference for the anatomical coordinate system was available. Furthermore, there was no correction for the orientation of the brain. Although all patients were imaged head first supine, there is some variance in the posture. Finally, the number of training examples for several of the ROIs were very small (see Table 1), and it is very likely that a larger dataset with more examples would improve the results.

The networks presented in this paper all relied on two‐dimensional data, or a series of two‐dimensional images as inputs. While it would certainly be possible to use a network that utilizes three‐dimensional data and performs classification on the whole structure, this would require a network with a larger memory footprint, and it was decided for the purpose of this study to limit the effort to using a series of two‐dimensional images and the voting mechanism as a sort of proxy for three‐dimensional classification.

## 5 CONCLUSION

We have effectively demonstrated a robust algorithm for the classification of anatomical structures in head and neck patients in a radiation oncology setting. We have also demonstrated how normalization of feature data and including data from multiple sources impacts the quality of classification. These algorithms are capable of classification of numerous structures with mean accuracy per class of 97.6% for the nonanatomically normalized algorithm, and 97.9% for the brain anatomically normalized algorithm, and 96.7% mean accuracy per class for the eye‐brainstem anatomically normalized algorithm. Total accuracy of the nonanatomically normalized algorithm achieved 99.0% accuracy, while the brain anatomically normalized algorithm achieved 98.3% total accuracy, and the eyes‐brainstem algorithm achieved a total accuracy of 97.9%.

## AUTHORS' CONTRIBUTION

David Livermore: Data Curation, InvestigationMethodology (equal), Software, Writing ‐ Original Draft Preparation (lead). Alasdair Syme: Conceptualization, Methodology (equal), Project Administration, Supervision (equal), Validation (equal), Writing ‐ Original Draft Preparation, Writing ‐ Review & Editing (lead). Thomas Trappenberg: Methodology (equal), Resources, Supervision (equal), Validation (equal), Writing ‐ Review & Editing

## CONFLICT OF INTEREST

The authors have declared no conflict of interest.
